# Neuronal GHS-R Differentially Modulates Feeding Patterns under Normal and Obesogenic Conditions

**DOI:** 10.3390/biom12020293

**Published:** 2022-02-11

**Authors:** Jong Han Lee, Bingzhong Xue, Zheng Chen, Yuxiang Sun

**Affiliations:** 1 Department of Marine Bio and Medical Science, Hanseo University, Seosan 31962, Korea; jhleecw@gmail.com; 2 USDA/ARS Children’s Nutrition Research Center, Department of Pediatrics, Baylor College of Medicine, Houston, TX 77030, USA; 3 Department of Biology, Georgia State University, Atlanta, GA 30303, USA; bxue@gsu.edu; 4 Department of Biochemistry and Molecular Biology, The University of Texas Health Science Center at Houston, Houston, TX 77030, USA; zheng.chen.1@uth.tmc.edu; 5 Department of Nutrition, Texas A&M University, College Station, TX 7743, USA

**Keywords:** ghrelin, growth hormone secretagogue receptor (GHS-R), diet-induced obesity, feeding behaviors, meal size, meal duration, meal frequency

## Abstract

The orexigenic hormone ghrelin increases food intake and promotes obesity through its receptor, growth hormone secretagogue receptor (GHS-R). We previously reported two neuron-specific GHS-R knockout mouse lines, namely pan-neuronal deletion by Syn1-cre and hypothalamic deletion by AgRP-cre, exhibiting differential diet-dependent effects on body weight. GHS-R deficiency in neurons elicited less pronounced metabolic effects under regular diet (RD) than high fat diet (HFD). While there was no difference in total food intake of HFD in either mouse line, Syn1-cre; Ghsr*^f/f^* mice showed much greater anti-obesity effect than that of AgRP-cre; Ghsr*^f/f^* mice. Meal feeding pattern is known to have a major impact on energy homeostasis and obesity development. Here, we investigated the feeding behaviors of these two neuron-specific GHS-R knockout mice under RD and HFD feeding, by assessing meal number, meal size, meal duration, and feeding frequency. Under the normal diet, RD-fed Syn1-cre; Ghsr*^f/f^* mice showed a decreased meal size in dark phase, while RD-fed AgRP-cre; Ghsr*^f/f^* mice showed an increased meal duration in dark phase. Under the obesogenic diet, HFD-fed Syn1-cre; Ghsr*^f/f^* mice displayed reduced meal numbers in light phase and increased feeding in both light and dark phases, whereas HFD-fed AgRP-cre; Ghsr*^f/f^* mice showed a decreased meal duration in the light phase only. Consistently, the expression of neuropeptides (Neuropeptide Y and Orexin) was increased in the hypothalamus of RD-fed Syn1-cre; Ghsr*^f/f^* mice, whereas the expression of cannabinoid receptor type 1 (CB1) was increased in the hypothalamus of HFD fed Syn1-cre; Ghsr*^f/f^* mice. Overall, feeding pattern changes were more pronounced in Syn1-cre; Ghsr*^f/f^* mice than that in AgRP-cre; Ghsr*^f/f^* mice, and HFD elicited greater alteration than RD. While AgRP-cre; Ghsr*^f/f^* mice consumed HFD meals faster during the day (showing shorter meal duration), Syn1-cre; Ghsr*^f/f^* mice ate few HFD meals during the light phase and ate slowly throughout the day (showing longer meal duration in both phases). Our findings reveal that neuronal GHS-R regulates energy homeostasis by altering feeding patterns, and differentially modulates feeding patterns in a site- and diet-dependent manner. The distinctive data in these two mouse lines also suggest that eating slowly during the optimal feeding period (dark phase for mice) may be beneficial in combating obesity.

## 1. Introduction

Obesity is a complex disease characterized by excess accumulation of body fat [1]. Changes in diet composition, feeding behaviors and physical activity are all implicated in the development of obesity [2,3,4]. In particular meal pattern, including meal size, feeding frequency and eating duration, has been shown to directly/indirectly affect body weight gain [5]. Moreover, it has been shown that meal size and frequency affect neuronal plasticity and vulnerability to metabolic diseases such as obesity, insulin resistance and diabetes [5]. 

Ghrelin is an orexigenic hormone secreted from the stomach. It stimulates appetite and increases food consumption in humans and rodents through its receptor, growth hormone secretagogue receptor (GHS-R) [6,7]. Plasma ghrelin levels increase before meals and rapidly decline after food intake [8]. While ghrelin plays an important role in the regulation of energy homeostasis [9], we and others have shown that deletion of ghrelin and/or GHS-R does not change total daily food intake and body weight in mice fed a standard diet [10,11,12]. Intriguingly, central ghrelin administration has been shown to alter meal patterns such as feeding frequency and meal size [13]. Consistently, we found that global Ghsr knockout mice have increased average meal size and feeding duration but decreased meal frequency, with total food intake unaffected [14]. While these results strongly suggest a role of GHS-R in meal pattern regulation, questions remain in regard to what brain regions are involved and whether metabolic condition affects the feeding pattern outcome. 

In the present study, to further elucidate the role of ghrelin signaling on meal pattern, we assessed the meal size, meal duration and feeding frequency of pan-neuronal GHS-R knockout mice (Syn1-cre; Ghsr*^f/f^*) and hypothalamic AgRP specific GHS-R knockout mice under both normal RD and obesogenic HFD feedings. Our data showed that neuronal GHS-R plays an important role in the control of eating patterns by coordinated regulation of the orexigenic and anorexigenic signals in a brain region- and diet-dependent manor.

## 2. Materials and Methods

### 2.1. Mice

The Syn1-Cre; Ghsr*^f/f^* and AgRP-cre; Ghsr*^f/f^* mice were generated as in our previous reports [15,16]. Age-matched male Ghsr*^f/f^* and Syn1-Cre; Ghsr*^f/f^* or AgRP-cre; Ghsr*^f/f^* mice were fed either regular diet (RD) or high-fat diet (HFD). The HFD used was Teklad (TD. 88137) with the composition of 42% calories from fat, 42.7% calories from carbohydrate, and 15.2% calories from protein. Mice were housed in a pathogen-free facility, at ambient temperature of ∼23 ± 1 °C, with 12-h light/dark phases. The age of mice used in the experiments: RD-fed Syn1-Cre; Ghsr*^f/f^* mice were 4.5 months old, HFD-fed Syn1-Cre; Ghsr*^f/f^* mice were 4.5 months old, RD-fed AgRP-Ghsr*^f/f^* mice were 9 months old, and HFD-fed AgRP-Ghsr*^f/f^* mice were 11 months old. All experimental procedures were approved by the institutional animal care and use committee of Baylor College of Medicine. 

### 2.2. Meal Pattern

Male Syn1-Cre; Ghsr*^f/f^* or AgRP-cre; Ghsr*^f/f^* mice and their age-matched Ghsr*^f/f^* littermates were individually housed and given free access to either RD or HFD and water as described previously [15,16]. After acclimatization, the CLAMS Comprehensive Laboratory Animal Monitoring System (Columbus Instruments, Columbus, OH) was used to monitor food intake and meal patterns. All data were analyzed as described in our previous published paper [14]. In brief, a feeding bout was recorded when ≥0.02 g of food was consumed, and the balance was stable for ≥10 s. Meal size was defined with weight of food consumed during each feeding bout. The length of each feeding bout and number of feeding bouts were assessed daily. All data were averaged over the 72 h period, and the mean values were used to generate the data for each group.

### 2.3. Real-Time PCR

The hypothalamus was dissected following a previously described method in our published papers [15,16]. Micro-punched brain tissues were stored at −80 °C until use. Total RNA was isolated using TRIzol reagent (Invitrogen, Carlsbad, CA) or RNeasy Mini kit (Qiagen, Germantown, MD), and cDNA was synthesized with the SuperScript III First-Strand Synthesis System for RT-PCR (18080085; Invitrogen, Carlsbad, CA, USA). SYBR Green-based real-time PCR was performed using an ABI Prism 7900 sequence detection system (4364344; Applied Biosystems, Waltham, MA, USA). Relative gene expression was calculated after normalization to 18S genes as described previously [14]. All primer sequences used are available upon request. 

### 2.4. Statistical Analysis

All data were represented as the mean ± SEM. Tukey’s post hoc multiple comparison test was used to follow up the significant differences after one way ANOVA analysis. *p* < 0.05 was considered statistically significant.

## 3. Results

In our previous studies, we showed that body weight and fat percentage of Syn1-Cre; Ghsr*^f/f^* mice were slightly reduced under RD-feeding, but drastically reduced under HFD-feeding, as a matter of fact showing a total prevention of diet-induced obesity [15]. In comparison, while the body weight and fat percentage of AgRP-cre; Ghsr*^f/f^* mice were unaltered under normal RD feeding, body weight and fat were significantly decreased under HFD-feeding [16]. However, the degree of body weight and fat mass decrease in HFD-fed AgRP-cre; Ghsr*^f/f^* mice was much less pronounced than that in HFD-fed Syn1-Cre; Ghsr*^f/f^* mice, the later has a remarkable anti-obesity phenotype [15,16]. Important to note, there was no difference in total daily intake of RD or HFD between genotypes in either Syn1-Cre; Ghsr*^f/f^* and AgRP-cre; Ghsr*^f/f^* mice (Figure S1), similar to we reported previously [15,16]. To further determine whether neuronal Ghsr regulates feeding behaviors and identify the key GHS-R expressing neurons involved, we studied meal patterns of Syn1-Cre; Ghsr*^f/f^* and AgRP-cre; Ghsr*^f/f^* mice under RD and HFD feedings.

### 3.1. Meal Number

**Figure 1:**
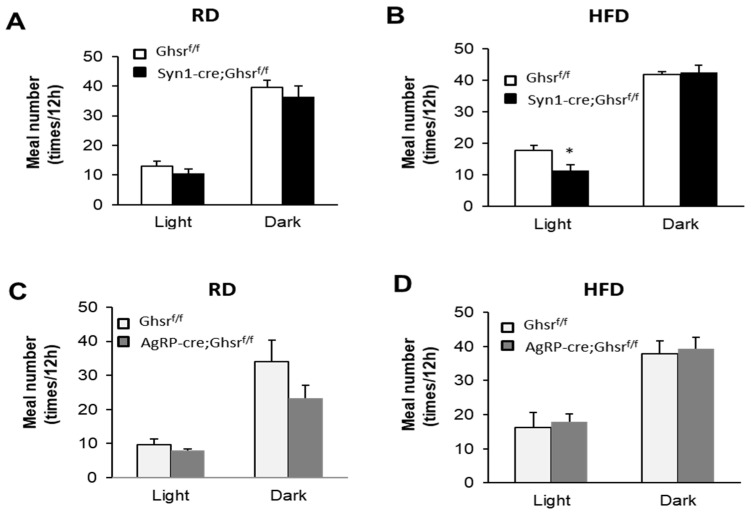
Meal number in Syn1-Cre; Ghsr*^f/f^* and AgRP-Ghsr*^f/f^* mice. RD (**A**,**C**) and HFD (**B**,**D**), *n* = 5, * *p* < 0.05, Ghsr*^f/f^* vs. Syn1-Cre; Ghsr*^f/f^* or AgRP-cre; Ghsr*^f/f^*. All data are presented as means ± SEM.

Under RD feeding, Syn1-Cre; Ghsr^f/f^ mice showed similar meal number when compared with control Ghsr^f/f^ mice (Figure 1A). Interestingly, under HFD feeding, Syn1-Cre; Ghsr^f/f^ mice displayed a significantly reduced meal numbers in the light phase but not in the dark phase (Figure 1B). In comparison, AgRP-cre; Ghsr^f/f^ mice showed a trend of lower meal number relative to controls under RD, but not under HFD (Figure 1C,D). 

### 3.2. Meal Size

**Figure 2:**
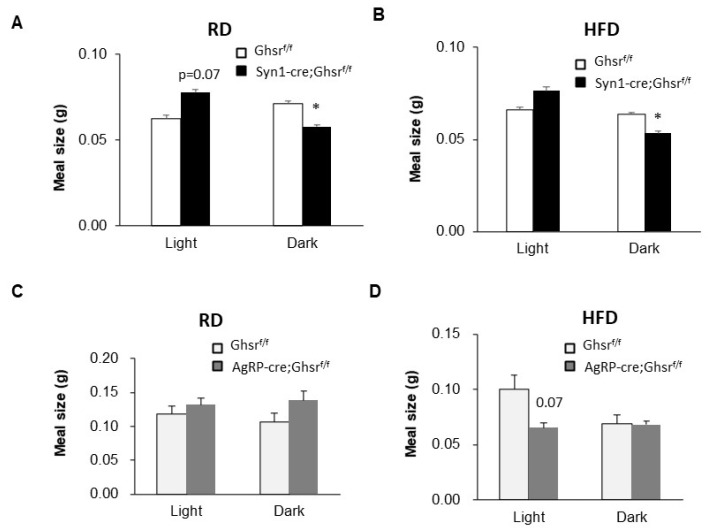
Meal size of Syn1-Cre; Ghsr*^f/f^* and AgRP-Cre; Ghsr*^f/f^* mice. RD (**A**,**C**) or HFD (**B**,**D**), *n* = 5, * *p* < 0.05, Ghsr*^f/f^* vs. Syn1-Cre; Ghsr*^f/f^* or AgRP-cre; Ghsr*^f/f^*. All data are presented as means ± SEM.

In both RD- and HFD-fed Syn1-Cre; Ghsr*^f/f^* mice, while the average meal size was modestly increased compared to that in Ghsr*^f/f^* in the light phase, it was significantly decreased in the dark phase (Figure 2A,B). At the same time, AgRP-cre; Ghsr*^f/f^* mice had no significant difference in meal size under RD-feeding (Figure 2C), but exhibited a clear trend of decrease (*p* = 0.07) under HFD-feeding during the light phase (Figure 2D). 

### 3.3. Meal Duration

**Figure 3:**
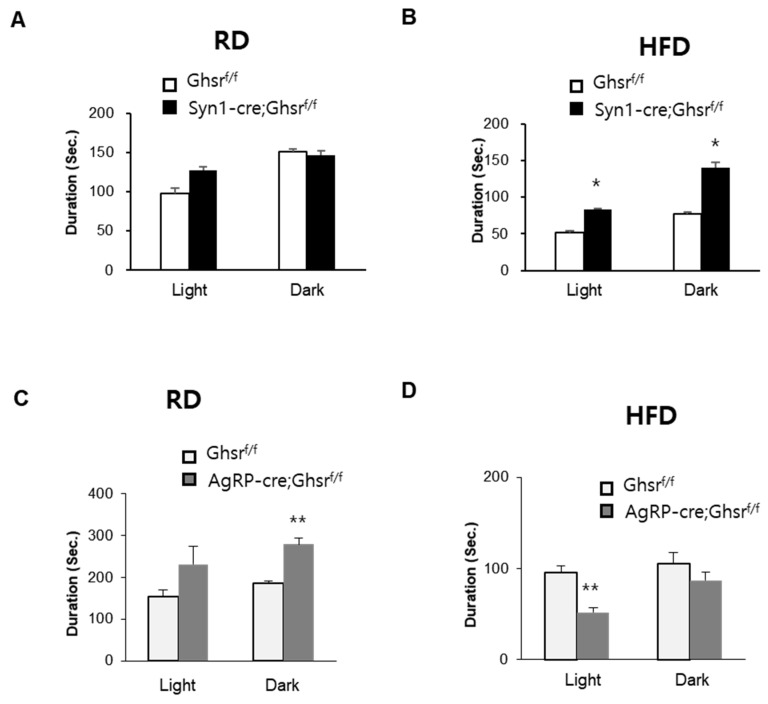
Duration in Syn1-Cre; Ghsr*^f/f^* and AgRP-cre; Ghsr*^f/f^* mice. RD (**A**,**C**) and HFD (**B**,**D**), *n* = 5, * *p* < 0.05, ** *p* < 0.01, Ghsr*^f/f^* vs. Syn1-Cre; Ghsr*^f/f^* or AgRP-cre; Ghsr*^f/f^*. All data are presented as means ± SEM.

We next analyzed the average feeding duration. In RD-fed Syn1-Cre; Ghsr*^f/f^* mice, the feeding duration revealed largely no difference (Figure 3A). In contrast, HFD-fed Syn1-Cre; Ghsr*^f/f^* mice showed a marked increase of feeding duration during both light and dark phases (Figure 3B). In RD-fed AgRP-cre; Ghsr*^f/f^* mice, the duration of feeding was significantly increased in the dark period (Figure 3C). In contrast, HFD-fed AgRP-cre; Ghsr*^f/f^* mice exhibited significantly decreased feeding duration in the light phase (Figure 3D). 

### 3.4. Neuropeptide Changes

**Figure 4:**
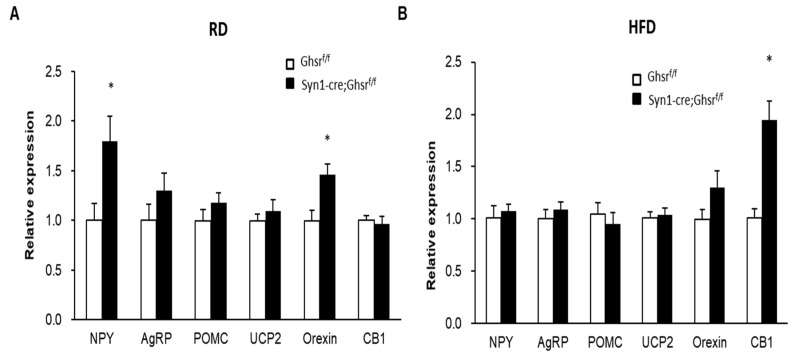
mRNA gene expression in the hypothlamsus of Syn1-Cre; Ghsr*^f/f^* and Ghsr*^f/f^* mice fed RD (**A**) and HFD (**B**). *n* = 5, * *p* < 0.05, Ghsr*^f/f^* vs. Syn1-Cre; Ghsr*^f/f^*. All data are presented as means ± SEM.

Orexigenic neuropeptide Y (NPY) and agouti-related peptide (AgRP) are known to stimulate appetite and enhance feeding, while anorexic neuropeptide Pro-opiomelanocortin (POMC) is known to suppress them [17]. Since GHS-R is highly expressed in NPY/AgRP neurons, but not in POMC neurons, we investigated whether these hypothalamic neuropeptides are concomitant with the altered meal patterns observed in these mice. As expected, the expression of orexigenic NPY in hypothalamus was increased in RD-fed Syn1-Cre; Ghsr*^f/f^* mice, while the expression of anorexic POMC remained unchanged (Figure 4A). Orexin is another neuronal peptide that is known to regulate feeding and sleep; similar to NPY, the expression of orexin was increased in RD-fed Syn1-Cre; Ghsr*^f/f^* mice (Figure 4A). CB1, a cannabinoid receptor, is known to simulate food intake and modulate reward, and CB1 has been shown to mediate the signal activation of GHS-R [18]. Interestingly, we found that CB1 expression was increased in the hypothalamus of HFD-fed Syn1-Cre; Ghsr*^f/f^* mice, while the expression of other genes tested was similar under HFD feeding (Figure 4B). 

We previously reported that HFD-fed AgRP-cre; Ghsr*^f/f^* mice have increased AgRP expression, but no difference in NPY expression [16]. The gene expression profile of AgRP-cre; Ghsr*^f/f^* mice is different from that of Syn1-Cre; Ghsr*^f/f^* mice shown in Figure 4. 

## 4. Discussion

We previously reported that global Ghsr knockout mice have increased meal size and feeding duration but decreased meal frequency [14]. In the present study, we assessed the feeding patterns of pan-neuronal Syn 1-specific and hypothalamic AgRP-specific GHS-R knockout mice. Syn1-Cre; Ghsr*^f/f^* mice ate smaller RD meals in dark phase, but fewer HFD meals in light phase with longer feeding duration during both light and dark phases. In contrast, AgRP-cre; Ghsr*^f/f^* mice showed increased feeding duration of RD meals in dark phase, but decreased feeding duration of HFD meals in light phase. Overall, the feeding behavior phenotype was more pronounced in Syn1-cre; Ghsr*^f/f^* mice than in AgRP-cre; Ghsr*^f/f^* mice. Our data collectively illustrate that GHS-R in different neurons have differential effects on feeding behaviors, which is diet-dependent in nature. Our findings, that the meal pattern changes were more pronounced under HFD than RD, suggest that GHS-R has an important role in regulation of feeding behavior under obesogenic condition.

Neuropeptides are integral part of the molecular mechanism underpinning feeding behaviors. The melanocortin system, such as NPY/AgRP and POMC expressing neurons, is one of the most important neuronal pathways involved in the regulation of food intake [19,20]. Ghrelin mediates hunger sensation and induces meal initiation through NPY and AgRP in the hypothalamic arcuate nucleus [21,22,23]. We previously reported that hypothalamic NPY and AgRP expression was increased, but POMC expression was decreased in RD-fed old global Ghsr knockout mice, with no difference in young mice [14]. This gene expression profile suggests that GHS-R may alter melanocortin system to modulate feeding patterns, which may explain the increased meal size and feeding duration observed in global GHS-R KO mice [14]. In our current study, under RD feeding, hypothalamic orexigenic NPY and orexin expression was increased, but anorexic POMC was similar in RD-fed Syn1-Cre; Ghsr *^f/f^* mice. Under HFD feeding, both orexigenic and anorexigenic gene expression was unaltered, but the appetite and reward regulator CB1 was elevated. In the current study, we found that AgRP-cre; Ghsr*^f/f^* mice exhibiting a different feeding pattern than that of Syn1-Cre; Ghsr *^f/f^* mice under both diets. In HFD-fed AgRP-cre; Ghsr*^f/f^* mice, we previously reported that orexigenic AgRP expression was increased [16]. GHS-R induced meal pattern changes may be mediated by orexigenic and anorexic neuropeptides in different brain regions that differentially respond to different diets. The differential feeding patterns exhibited by Syn1-Cre; Ghsr *^f/f^* and AgRP-cre; Ghsr*^f/f^* mice may be due to a direct effect on the hypothalamic orexigenic NPY/AgRP pathway, an indirect effect on anorexigenic POMC pathway, and/or an effect on GHS-R downstream mediators such as CB1. 

HFD consumption alters a variety of behaviors independent of obesity [24]. Studies have shown that obesity triggers broad dysregulation of hypothalamic hunger neurons, and these alterations are specifically responsive to dietary fat [25]. We previously reported that in HFD-fed AgRP-specific Ghsr KO mice, AgRP expression was significantly increased, while anorexic neuropeptide POMC expression was modestly decreased, suggesting that the activation of orexigenic signal rather than suppression of anorexigenic signal drive the feeding pattern of AgRP-Cre; Ghsr*^f/f^* mice [16]. CB1 expression in hypothalamus is associated with altered feeding behavior [26,27,28] and its expression is highly affected by HFD [29]. Our current study showed that CB1 expression is increased in HFD-fed Syn1-Cre; Ghsr*^f/f^* mice, while other melanocortin genes were unaffected. This suggests that melanocortin system likely does not play a dominant role in defining feeding patterns of Syn1-Cre; Ghsr*^f/f^* mice under obesogenic condition, instead other signaling pathways, such as CB1, may play a dominant role in GHS-R mediated feeing behavior moderation. Taken together, these data suggest that GHS-R regulates feeding behaviors, likely by modulating hypothalamic peptides, which is dependent on metabolic condition and involves regulating pathways beyond the melanocortin system. 

Eating habits influence body weight gain and body composition changes [30,31,32]. We previously reported that while there was remarkable anti-obesity phenotype in HFD-fed Syn1-Cre; Ghsr*^f/f^* and AgRP-cre; Ghsr*^f/f^* mice, the total daily food intake was no different [15,16]. Ghrelin is known to regulate satiety by reducing gastric vagal afferents, making it less susceptible to bloating sensation of overeating [33]. Therefore, we anticipate that GHS-R activation possibly induces meal size and duration. Interestingly, RD-fed AgRP-cre mice showed increased feeding duration in dark phase, similar to our previous observations in global Ghsr KO mice [14]. Others have reported that HFD feeding changes feeding pattern and preference for meal size [34,35]. Indeed, we found that HFD-fed AgRP-cre mice exhibited decreased feeding duration in the light phase, whereas HFD fed Syn1-cre; Ghsr*^f/f^* mice displayed decreased meal number in light phase and increased feeding duration throughout the day. Mice are nocturnal animals, they consume food mostly at dark phase (the primary feeding period). Intriguingly, our data showed that there was no difference in the meal numbers at the dark phase regardless of food types in both mouse models, but exhibited differential meal size and duration in these GHS-R neuronal knockout models. While AgRP-cre; Ghsr*^f/f^* mice ate HFD meals faster during the light phase (showing shorter meal duration), Syn1-cre; Ghsr*^f/f^* mice ate few HFD meals during the light phase and ate slowly throughout the day (showing longer meal duration in both phases). Considering HFD-fed Syn1-cre; Ghsr*^f/f^* mice have more pronounced anti-obesity phenotypes than AgRP-cre; Ghsr*^f/f^* mice, the distinctive feeding patterns suggest that eating slowly during the optimal feeding period (dark phase for mice) may be beneficial in combating obesity.

In addition to its orexigenic effect, the endocannabinoid signaling in the brain is known to be involved in regulation of rewards such as preferences of palatable food and addictive drugs [36]. Cannabinoids that are lipid messengers functionally interact with neurotransmitters in neural networks to control feeding behavior [37]. CB1, a hallmark cannabinoid receptor, is expressed in various brain regions involved in reward and addiction, including the basolateral amygdala, substantia nigra (SN), ventral tegmental area (VTA), prefrontal cortex, ventral pallidum, hippocampus, globus pallidus, dorsolateral striatum, central nucleus of the amygdala and bed nucleus of the stria terminalis [36,38]. CB1 is localized in glutamatergic synapses of dopaminergic VTA neurons. CB1 plays an important role in fine-tuning activity of dopaminergic projections by modulating the excitatory and inhibitory signals in the VTA [39,40]. Interestingly, it has been shown that CB1 mediates signal activation of GHS-R [18]. Ghrelin increases hypothalamic endocannabinoid content in control mice, but not in CB1 KO and CB1 receptor antagonist rimonabant-treated mice; moreover, the effects of ghrelin on food intake and AMPK activation are ablated in CB1 KO and rimonabant-treated mice [41]. These results suggest that an intact cannabinoid signaling is required for ghrelin’s activation of AMPK. We previously showed that GHS-R has differential effects in VTA and SN neurons [15]. Ghsr deletion in VTA may reduce dopamine reuptake and suppress dopamine turnover, thus reducing reward- and motivation-associated responses. We have also showed that GHS-R in AgRP neurons is involved in neurocircuitry of metabolic adaptation under calorie restriction condition [42]. Based on these findings, we postulate that there is crosstalk between nutrient sensing ghrelin-GHS-R and reward CB1 signaling, they reciprocally sense energy state and modulate reward circuit to alter feeding behaviors in response to various metabolic cues such as overnutrition or energy-deficiency. More in-depth investigation is needed to verify this putative network between the energy sensing ghrelin-GHS-R signaling and the reward CB1 circuitry. 

In conclusion, our study uncovered an important new aspect of ghrelin biology that in addition to its well-known orexigenic effect, ghrelin signaling also regulates energy metabolism by modulating feeding behaviors. Our data showed that GHS-R plays an important role in regulation of feeding patterns, and neural GHS-R regulates feeding patterns in a site-dependent and diet-dependent manner. GHS-R likely fine-tune orexigenic and anorexigenic signals in the brain to modulate feeding patterns, thereby regulating overall energy homeostasis. 

## Data Availability

Not applicable.

## Supplementary Materials

The following supporting information can be downloaded at: https://www.mdpi.com/article/10.3390/biom12020293/s1, Figure S1: Total food intake in Syn1-cre; Ghsr*^f/f^* and AgRP-Ghsr*^f/f^* mice. RD (A,C) and HFD (B,D), *n* = 5, All data are presented as means ± SEM.
