# Supplementary material for: Neuronal GHS-R Differentially Modulates Feeding Patterns under Normal and Obesogenic Conditions
Source: Biomolecules. 2022 Feb 11;12(2):293. doi: 10.3390/biom12020293 (PMC8961776; doi:10.3390/biom12020293)
Supplement: Supplementary file 1 [file biomolecules-12-00293-s001.zip › biomolecules-1574837-supplementary.pdf]

Supplementary Figure S1

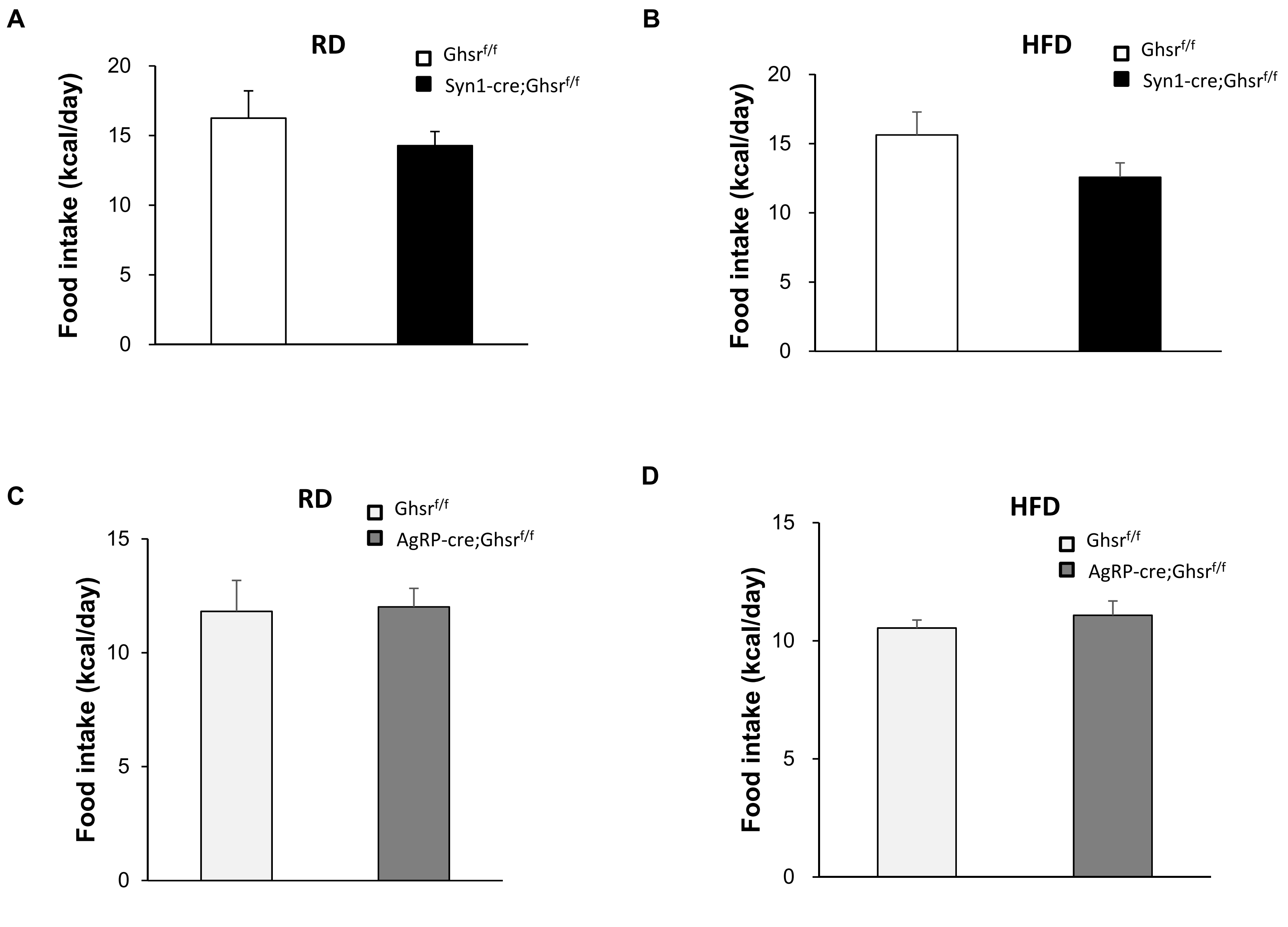

Figure S1. Total food intake in Syn1-Cre;Ghsrf/f and AgRP-Ghsrf/f mice. RD (**A**, **C**) and HFD (**B**, **D**), n=5, All data are presented as means ± SEM.
